# The contribution of family medicine to community-orientated health services in Mali: A short report

**DOI:** 10.4102/phcfm.v13i1.3047

**Published:** 2021-09-30

**Authors:** Mahamane M. Maïga, Gabriel Blouin Genest, François Couturier, Sarah Stecko, Michèle Rietmann, Mamadou B. Coulibaly, Drissa M. Sidibé, Fatoumata Dicko

**Affiliations:** 1 Department of Family Medicine, Faculty of Medicine and Health Sciences, Université de Sherbrooke, Sherbrooke, Canada; 2 School of Applied Politics, Faculty of Arts and Humanities, Université de Sherbrooke, Sherbrooke, Canada; 3 Department of Administration, Faculty of Medicine and Health Sciences, Université de Sherbrooke, Sherbrooke, Canada; 4 University Community Health Center of Konobougou, Konobougou, Mali; 5 Department of Family and Community Medicine, Université des Sciences, Des Techniques et des Technologies de Bamako (U.S.T.T.B), Bamako, Mali

**Keywords:** family medicine, decentralisation, training, partnership, community, women and girls, multidisciplinary, primary health care

## Abstract

Family medicine has not received appropriate attention in the sub-Saharan African context. In particular, family medicine is rarely recognised as a medical speciality and most African countries are silent on the role of family medicine in their health systems. There is, however, an emerging interest in developing family medicine as a key component of primary healthcare. Postgraduate training in family medicine is progressing and many countries have already established specific training programmes. In addition, there have been attempts to define the importance of family medicine, which, we expect, this short report contributes to. Interviews were conducted with physicians, partners and beneficiaries of two international development projects funded by the Canadian government. The one project supports training of health professionals and the other education of healthy women and girls in the community. The objective was to document the strengthening of primary healthcare through the creation and adaptation of a new family and community medicine postgraduate medical programme (which includes both family and community medicine) emphasising field training, immersion in local communities and interdisciplinary collaboration. This article underlines the importance of family medicine in Mali by documenting how what is now termed family and community medicine can promote community-orientated health services. To do so, we use the examples of initiatives and actions done through two international health development projects.

## Introduction

The World Health Organization (WHO) recommends different strategies to strengthen what they call ‘integrated people-centred health services’.^1^ One strategy is to re-orientate the health system towards strong primary healthcare that includes family medicine. This approach has not received appropriate attention in sub-Saharan Africa.^2^ Furthermore, the role of family medicine and its impact on health services and communities has not been adequately documented. The role of family medicine is especially important in low-resource contexts where a heavy burden of disease is experienced. Because of the lack of resources, healthcare in many African countries relies primarily on donor-driven and vertical disease-orientated programmes.^3^ The role of family medicine is poorly understood^4^ and the discipline is rarely recognised as a medical speciality. Most African countries are silent on the role of family medicine in their health systems^4^ and priority is given to hospital-centred services, a phenomenon amplified in rural areas.^3^

There is, however, an emerging interest in developing family medicine as a key component of district health services in the sub-Saharan African context. Postgraduate training in family medicine is progressing and several countries have established training programmes.^3^ In addition, there have been attempts to define the importance of family medicine, as seen for example with the consensus statement on family medicine during the African Regional WONCA Conference in 2009.^2^ This short report seeks to add to this by documenting how what is now termed ‘family and community medicine’, combining both family physician training and a community health approach, can promote community-orientated health services in Mali.

Interviews were conducted with physicians, partners and beneficiaries of two international development projects, namely, DECLIC (*Projet d’appui à la formation des professionnels de la santé au Mali*), a support project for the training of health professionals, and CLEFS (*Communautés locales d’enseignement pour les femmes et les filles en santé*), a local education initiative for healthy women and girls (Projects financed by Global Affairs Canada, Canada’s Department of Foreign Affairs, Trade and Development; in effect since 2012). These projects strengthen the primary healthcare through the creation and adaptation of a new family and community medicine postgraduate medical programme, by emphasising field training, immersion in local communities and interdisciplinary collaboration. By linking the training of health professionals with the needs of the population, these projects make the advanced services to reach the communities through local university-related health clinics.

## Bringing family medicine to those in need through community-based clinics

Mali’s health system is highly decentralised (compared with neighbouring countries) and is built on a pyramidal structure with the first level being the Community Health Centre (CSCom). The CSCom is a health institution dating from the end of the 1990s with a mission to provide public health services in a specific socio-sanitary area. The CSCom is managed by community stakeholders through an association known as *Associations de santé communautaire*, or ASACO. Community Health Centres promote community participation in the management of individual and community health issues. Community Health Centres are the centrepiece of Malian national health policies, playing an even more extensive role in rural areas, given the difficulties in accessing health services in these areas.

Mali has also enacted University Community Health Centres (CSCom-U), adding university accreditation to CSComs to adequately train health professionals and reach rural/remote areas. These centres are accredited by the combined Faculty of Medicine and Odontostomatology (FMOS; Bamako University of Sciences, Techniques and Technologies) and perform clinical training, education and research for family and community medicine students, midwives, nurses and laboratory technicians, thereby underlining the importance of interdisciplinary teamwork focusing on primary care.

It is an innovative model in French-speaking West Africa, but one that has been proven successful elsewhere in the world.^5^ As mentioned by one interviewee, ‘family and community medicine did not exist before in Mali. This is the innovation brought in by these projects’ (Doctor and professor, male, 2018). A single new family and community medical postgraduate programme was created in 2012, including an objective to decentralise training, where the students are deployed in the distributed platform, close to the population, during three out of the 4 years of their training. Family physicians can now be trained directly in rural and remote areas through the CSCom-U network, better responding to the needs of communities and involving them in the planning and organisation of the different health services.

‘The university is moving out of the big cities to the outlying places,’ one professor mentioned (Professor, male, 2018). ‘It’s appropriate because it’s their [*the trained health professionals*] future place of work’ (Professor, male, 2018). This helps respond to the challenge of reaching those living in remote areas:

‘In our community, there is a real problem with regard to the care of people in remote areas. Quality care is essentially in the big cities, but we know that the majority of the population is elsewhere.’ (Doctor, male, 2018)

As a result, family and community medicine physicians are better able to respond adequately to the needs of the populations, whilst contributing to better training of front-line professionals and bringing knowledge to patients and users. This community-orientated approach of family medicine is key to the appropriation and acceptance of health services by the users of these services:

‘Those who respond the most are the communities, underlined one professor, because they are in need. We are always in a community approach, negotiating with them […] and even make them plan the activities that they want to be carried out.’ (Doctor, male, 2018)

One woman said, for example, that before she came to CSCom-U, she knew that newborns needed to be vaccinated, but that now she says she understands ‘why it is important’, thanks to the availability and accessibility of an interdisciplinary team.

### Continuous quality improvement and the training of health professionals

Improving the quality of family medicine services and training is key to building strong and resilient community-orientated health services. Quality assessment evaluation (meaning the traditional quality evaluation of services) has been replaced with continuous quality improvement, which is a more dynamic approach. This includes community-orientated teaching methods and continuous improvement of services through community collaboration and applied research projects. Most of these research projects, which are conducted during the fourth year of the programme, use an action research methodology, by adding various community stakeholders in project development, data collection, result analysis and implementation of corrective measures, with the objective of strengthening social cohesion around primary care and family medicine. For example:

‘Amadou’s [*name changed*] action research [*on the reception of beneficiaries in the clinic’s reception area*] […] allowed the staff to express their concerns freely […] and it also allowed the patients to ask questions […]. It also shortened the waiting time, which was a big concern […]. The reception improved thanks to the action plan.’ (Doctor, male, 2018)

The projects also enabled educational institutions to become more open to pedagogical reforms. This includes the development of programmes based on community needs, training of students in close contact with the population served, field teaching and the focus of teaching on the student’s capacity and openness to conversation with the population: ‘What DECLIC has done, a physician said, is to improve the quality of care for the population. The quality of life of the population is bound to improve’ (Doctor, male, 2018).

The influence of the new family and community medicine programme also demonstrates that early exposure of health science students to primary healthcare and the concept of social responsibility is possible and productive as it promotes learner involvement with vulnerable and/or remote populations. A midwife student who was interviewed stressed, for example, that:

‘What we have never done, we don’t know how to do; we have only been given theory. […] So, when we went to the training sites, it was easier for us, it was like we were already there because we had already practised the interventions.’ (Midwife student, second year, female, 2018)

## Conclusion

Being distributed throughout the region, thanks to the CSComs network, family and community medicine physicians are now essential elements for improving primary health care through community-orientated health services. This has laid a solid foundation for significant change in the Malian health system, with already noticeable advances in the health of the Malian population, specifically women, girls and children.
